# PICO entity extraction for preclinical animal literature

**DOI:** 10.1186/s13643-022-02074-4

**Published:** 2022-09-30

**Authors:** Qianying Wang, Jing Liao, Mirella Lapata, Malcolm Macleod

**Affiliations:** 1 CCBS, Edinburgh Medical School, University of Edinburgh, Edinburgh, UK; 2 ILCC, School of Informatics, University of Edinburgh, Edinburgh, UK

**Keywords:** PICO, Preclinical animal study, Named entity recognition, Information extraction, Self-training

## Abstract

**Background:**

Natural language processing could assist multiple tasks in systematic reviews to reduce workflow, including the extraction of PICO elements such as study populations, interventions, comparators and outcomes. The PICO framework provides a basis for the retrieval and selection for inclusion of evidence relevant to a specific systematic review question, and automatic approaches to PICO extraction have been developed particularly for reviews of clinical trial findings. Considering the difference between preclinical animal studies and clinical trials, developing separate approaches is necessary. Facilitating preclinical systematic reviews will inform the translation from preclinical to clinical research.

**Methods:**

We randomly selected 400 abstracts from the PubMed Central Open Access database which described in vivo animal research and manually annotated these with PICO phrases for Species, Strain, methods of Induction of disease model, Intervention, Comparator and Outcome. We developed a two-stage workflow for preclinical PICO extraction. Firstly we fine-tuned BERT with different pre-trained modules for PICO sentence classification. Then, after removing the text irrelevant to PICO features, we explored LSTM-, CRF- and BERT-based models for PICO entity recognition. We also explored a self-training approach because of the small training corpus.

**Results:**

For PICO sentence classification, BERT models using all pre-trained modules achieved an F1 score of over 80%, and models pre-trained on PubMed abstracts achieved the highest F1 of 85%. For PICO entity recognition, fine-tuning BERT pre-trained on PubMed abstracts achieved an overall F1 of 71% and satisfactory F1 for Species (98%), Strain (70%), Intervention (70%) and Outcome (67%). The score of Induction and Comparator is less satisfactory, but F1 of Comparator can be improved to 50% by applying self-training.

**Conclusions:**

Our study indicates that of the approaches tested, BERT pre-trained on PubMed abstracts is the best for both PICO sentence classification and PICO entity recognition in the preclinical abstracts. Self-training yields better performance for identifying comparators and strains.

## Background

Systematic review attempts to collate all relevant evidence to provide a reliable summary of findings relevant to a pre-specified research question [1]. When conducting information extraction from clinical literature, the key elements of interest are Population/Problem, Intervention, Comparator and Outcome, which constitute the established framework of PICO [2]. This has been used as the basis for retrieval, inclusion and classification of published evidence, and empirical studies have shown the use of the PICO framework facilitates more complex search strategies and yields more precise search results in systematic reviews [3]. During citation screening, investigators screen the abstracts to determine the inclusion or exclusion of studies. Abstracts that are pre-structured according to the PICO frame or combined demonstration with PICO phrases enable faster judgement of study relevance for each PICO element [4]. Pre-structured PICO information also allows investigators to locate relevant descriptions from full-text articles which may speed up the data extraction process [5]. As the number of publications describing experimental studies has increased, the time taken in manually extracting information has increased such that many reviews are out of date by the time they are published. The evidence-based research community has responded by advocating the use of automated approaches to assist systematic reviews, and PICO extraction tools have been developed, particularly for clinical trials [6].

Preclinical animal studies differ from clinical trials in many aspects. The aim of animal studies is to explore new hypotheses for drug or treatment development, so they have more variations for the definition of PICO elements. For example, in animal studies, disease is not naturally present but often induced, different species can be used, and outcomes of interest can include survival, behavioural, histological and biochemical outcomes [7]. Considering the difference and the leading clinical research, the SYRCLE group developed a framework definition of preclinical PICO, where ‘Population’ includes animal species and strain and any method of inducing a disease model, and several outcomes can be considered [8]. Importantly, the ‘Comparator’ for animal studies is usually simply an untreated control cohort, although the exact choice of control is sometimes a variable of interest.

Here, we report the development of automatic PICO extraction approaches for preclinical animal studies which may advocate the use of preclinical PICO and facilitate the translation from preclinical to clinical research.

## Related work

To our knowledge, while automated PICO extraction in clinical reports is relatively well-explored, no method has been developed or evaluated for preclinical animal literature.

Most of the previous work for the clinical trial literature casts PICO element extraction as a sentence classification task. Byron et al. use logistic regression with distant supervision to train classifiers for PICO sentences derived from clinical articles [5]. More recent approaches have used recent neural networks for PICO sentence classification which requires less manual feature engineering. Such approaches include the bidirectional long-short term memory network (BiLSTM) [9] with some variations [9–11]. More precise PICO phrases or snippet extraction is cast as a named entity recognition task, and BiLSTM with conditional random field (CRF) [11] are common approaches [12–14]. Some advanced methods including graph learning [13] and BERT (a transformer-based machine learning model) [15] enhance the performance.

## Methods

### Dataset

We downloaded 2,207,654 articles from the PubMed Central Open Access Subset database^1^ published from 2010 to 2019 and used a citation screening filter trained to identify in vivo research from title and abstract (developed by EPPI-Centre, UCL [16]). We chose an inclusion cut point which gave 99% precision and obtained 50,653 abstracts describing in vivo animal experiments. We randomly selected 400 abstracts for the annotation task and another 10,000 for the self-training experiments.

We used the online platform tagtog^2^ for PICO phrase annotation. In addition to Intervention, Comparator and Outcome, we divided the Population category into three components: the Species, the Strain, and the method of Induction of the disease model. After the initial annotation process and discussion with a senior clinician, we proposed some general rules for the annotation task:

- Only PICO spans describing in vivo experiments are annotated, i.e. interventions or treatments should be conducted within an entire, living organism. Interventions applied to tissues derived from an animal or in cell culture (ex vivo or in vitro experiments) should not be annotated.
- Texts describing the introduction, conclusion or objectives should not be annotated in most cases because these might relate to work other than that described in the publication. They should be annotated only when the remaining text lacks a clear description of the method or where the text gives the meaning of abbreviations.
- The first occurrence of an abbreviation should be annotated together with the parent text. For example, ‘vascular endothelial growth factor (VEGF)’ should be tagged as one entity for its first occurrence; in the remainder of the text, ‘VEGF’ or ‘vascular endothelial growth factor’ could be annotated separately if they are not mentioned together.
- Any extra punctuations between phrases (such as commas) should not be annotated. However, if the entity appears only one time in the text, punctuations can be included in a long span of text which consists of several phrases.
- Entity spans cannot be overlapped. Annotations in tagtog are output in EntitiesTsv format which resembles the tab-separated values (tsv) output in the Stanford NER tool [17], and this does not support overlapping entities.

**Fig. 1:**
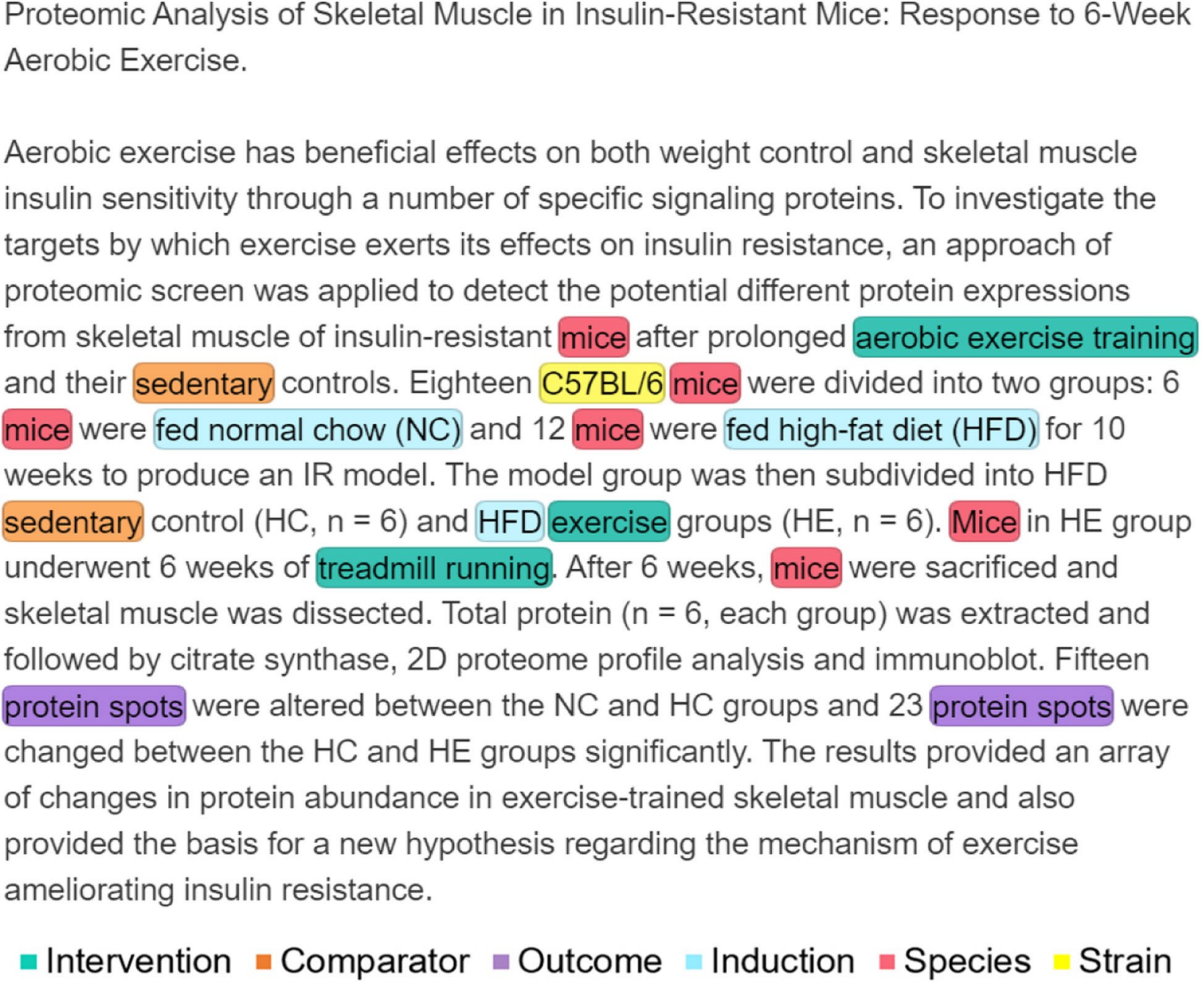
Preclinical PICO annotation example. Screenshot from tagtog

Figure 1 shows an example of annotated abstract using tagtog. After excluding the title, introduction sentence, first part of the objective sentence and the conclusion sentence which do not explicitly describe experimental elements, PICO entities are extracted from the remaining sentences: (1) Species: mice; (2) Strain: C57BL/6; (3) Induction: fed normal chow (NC), fed a high-fat diet (HFD); (4) Intervention: aerobic exercise training, exercise and treadmill running; (5) Comparator: sedentary; and (6) Outcome: protein spots.

In total, 6837 entities were annotated across 400 abstracts, and the distribution of PICO entities is imbalanced (Table 1). Less than 50% of sentences in each abstract contain PICO phrases, and using the entire abstracts to train an entity recognition model is not efficient. Therefore, we split the PICO phrase extraction task into two independent subtasks: (1) PICO sentence classification and (2) PICO entity recognition.

**Table 1:** Statistics of 400 annotated PICO dataset

**Average number in each abstract**	
PICO sentences	5
Sentences	11
Entities	17.5
**Distribution of PICO entity**	
Intervention	24.1%
Comparator	1.8%
Outcome	40.6%
Induction	10.6%
Species	19.6%
Strain	3.3%
Total	100%

### PICO sentence classification

Text from 400 abstracts are split into 4247 sentences by scispaCy [18], and sentences containing at least one PICO entity are labelled as ‘true’ for PICO sentence. Individual sentences were randomly allocated to training, validation and test sets (80%/10%/10%). For the sentence-level classification task, we use bidirectional encoder representation from transformers (BERT), a contextualised representation model where a deep bidirectional encoder is trained on a large text corpus. The encoder structure is derived from the powerful transformer based on multi-head self-attention, which dispenses with issues arising from recurrence and convolutions (an operation which applies sliding window functions on representation matrices to filter out information) [19]. The pre-trained BERT can be fine-tuned with a simple additional output layer for downstream tasks and achieves state-of-the-art performance on many natural language processing tasks [15]. We explore the effects of using different text corpora and methods for pre-training including (1) BERT-base, the original BERT trained on the combination of BookCorpus, and English Wikipedia [15]; (2) BioBERT, which trains BERT on the combination of BookCorpus, English Wikipedia, PubMed abstracts and PubMed Central full-text articles [20]; (3) PubMedBERT-abs, which trains BERT on PubMed abstracts only, and (4) PubMedBERT-full on a combination of PubMed abstracts and PubMed Central full-text articles [21].

The approach to training seeks to minimise cross-entropy loss (a loss function to evaluate the contrast between the predicted labels and true labels) using the AdamW algorithm [22]. We use a slanted triangular learning rate scheduler [23] with a maximum learning rate 5e−5 for 10 epochs of training. We apply gradient clipping [24] with a threshold norm of 0.1 to rescale gradients and gradient accumulation every 16 steps (mini-batches) to reduce memory consumption.

### PICO entity recognition

Identifying specific PICO phrases is cast as a named entity recognition (NER) task. We convert all entity annotations to the standard BIO format [25], i.e. each word/token is labelled as ‘B-XX’ if it is the beginning word of the ‘XX’ entity, ‘I-XX’ if it belongs to other words inside the entity but not the beginning word or ‘O’ if it is outside of any PICO entity. Hence, there are 13 unique tags for 6 PICO entities (two tags for each entity, plus tag ‘O’), and a NER model is trained to assign the 13 unique tags to each token in the PICO text.

One classic NER model is the bidirectional long-short term memory (BiLSTM) with a CRF layer on top (BiLSTM-CRF) [26]. LSTM belongs to the family of recurrent neural networks which can process word embeddings sequentially. In the hidden layer, by combining the weighted hidden representations from the adjacent word through a Tanh operation, a basic recurrent neural structure can retain information from neighbouring text. However, when the document is long, retraining information from very early or late words is difficult because of the exploding or vanishing gradient problem, which stops the network learning efficiently [27]. LSTM is designed to solve this long-term dependencies problem, which uses a cell state and three gates (forget gate, input gate and output gate) for each word embedding to control the information we need to flow straight, to forget or to store and update to the next step [9]. BiLSTM contains information from words in both directions, by processing hidden vectors from previous words to the current word and hidden vectors from future words back to the current words.

CRF is a type of discriminative probabilistic model which is often added on top of LSTMs to model dependencies and learn the transition constraints among predicted tags from LSTM output. For example, if the tag of a word in the sequence is ‘I-Outcome’, the tag of the previous word can only be ‘B-Outcome’ or ‘I-Outcome’, and impossible to be ‘I-Intervention’ or ‘O’ in a real sample. Models without the CRF layer may lose these constraints and cause unnecessary transition errors. We explore BiLSTM models with or without CRF layers. For text representations in these models, tokens are mapped into 200-dimension vectors by word2vec [28] induced on a combination of PubMed, PMC texts and English Wikipedia [29].

Similar to the PICO sentence classification, we also fine-tuned BERT with different pre-trained weights for the entity recognition task, using the BertForTokenClassification module from the Hugging Face Transformers library [30]. We also explored the effect of adding CRF and LSTM layers on top of BERT.

**Fig. 2:**
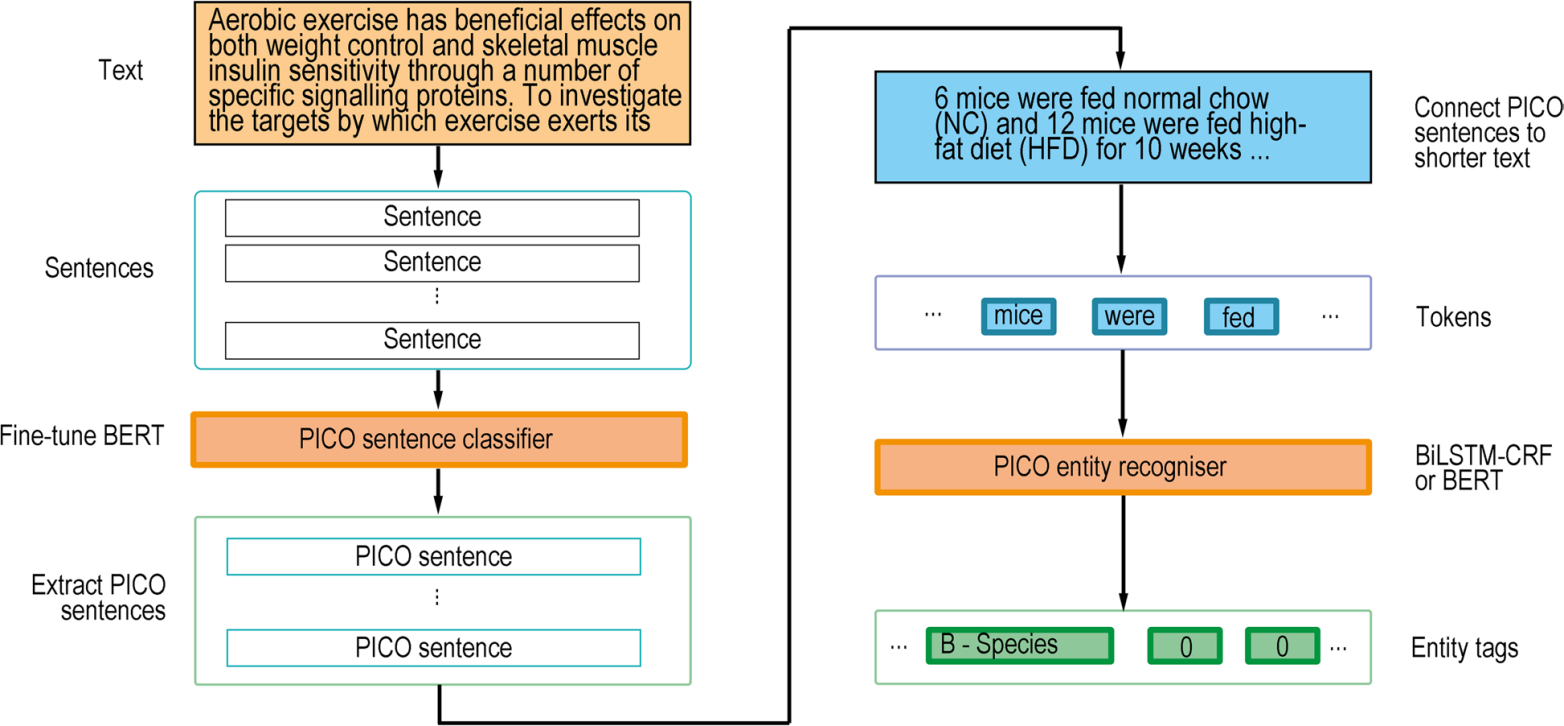
The workflow of the PICO extraction

For more efficient training and to achieve the best results for the entity recognition task, we removed the sentences without any PICO annotation from each abstract and trained NER models on each remaining text, which consisted of PICO sentences only; for prediction in the future application, sentences in an individual abstract are classified by the best PICO sentence classifier from the first task, and the non-PICO sentences are then removed automatically. The workflow is illustrated in Fig. 2.

For LSTM/CRF models, we tuned hidden dimensions from 32 to 512 and compare Adam and AdamW optimisers with the constant or slanted triangular learning rate scheduler. We froze word embeddings because we found it achieves better performance on the validation set. Models were trained for 20 epochs, and the learning rate depended on the specific model (1e−3 for BiLSTM and 5e−3 for BiLSTM-CRF). For BERT models, we fine-tuned BERT for 20 epochs with a learning rate of 1e−3, BERT-CRF for 30 epochs and BERT-LSTM-CRF for 60 epochs, both with a learning rate of 1e−4; other settings are similar to that of PICO sentence classification task. These settings were determined by checking overfitting or convergence issues from their learning curves. For evaluation, we used entity-level metrics [31] for each PICO text (truncated abstract):

$${\displaystyle \begin{array}{c}{\mathrm{Precision}}_i=\frac{\mathrm{number}\ \mathrm{of}\ \mathrm{predicted}\ \mathrm{correct}\ \mathrm{entities}}{\mathrm{number}\ \mathrm{of}\ \mathrm{predicted}\ \mathrm{entities}}\\ {}\begin{array}{c}{\mathrm{Recall}}_i=\frac{\mathrm{number}\ \mathrm{of}\ \mathrm{predicted}\ \mathrm{correct}\ \mathrm{entities}}{\mathrm{number}\ \mathrm{of}\ \mathrm{true}\ \mathrm{entities}}\\ {}\mathrm{F}{1}_{\mathrm{i}}=\frac{2\ast {\mathrm{Precision}}_i\ast {\mathrm{Recall}}_i}{{\mathrm{Precision}}_i+{\mathrm{Recall}}_i}\end{array}\end{array}}$$

These individual metrics were then averaged across all validation/test samples to obtain the overall metrics.

### Self-training

**Fig. 3:**
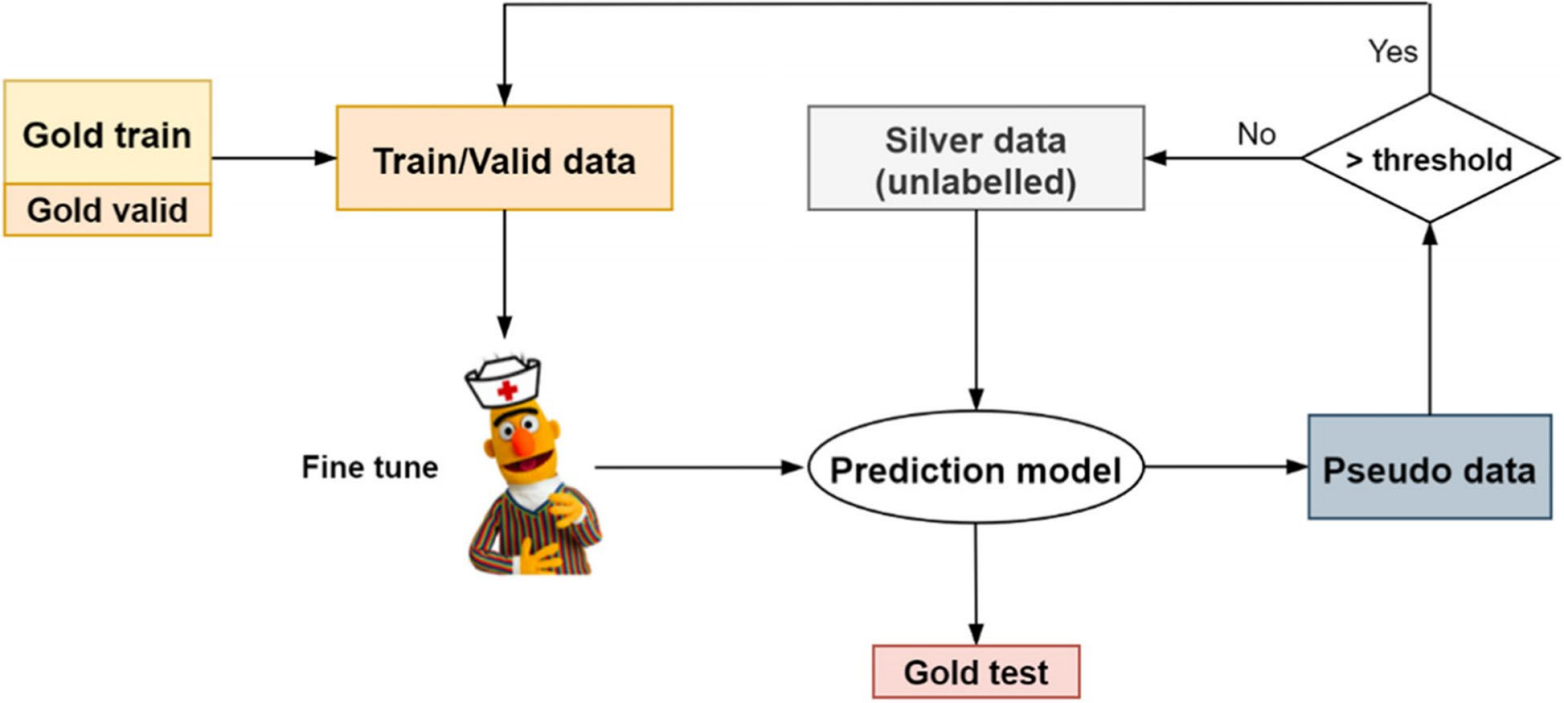
The workflow of the self-training in our experiments

One limitation of the previous method is the small amount of training data, so we also explored a semi-supervised learning strategy, self-training, which used the unlabelled dataset to generate pseudo labels for training [32]. We use 400 annotated abstracts as ‘gold’ data and 10,000 unlabelled abstracts from 50,653 in vivo animal records as ‘silver’ data. Non-PICO sentences were removed from the unlabelled text by the best PICO sentence classification model, and these truncated texts were used for self-training. As Fig. 3 shows, we first used the fine-tuned PICO entity recogniser from the gold set (80% of 400 labelled records for training, 10% for validation) to predict the entities of each token in the silver set. For each abstract in the silver set, we calculated the average prediction probabilities of all tokens within that abstract. Silver records with average probabilities larger than a threshold (0.95 or 0.99) were then combined with the original gold training/validation set, and the enlarged new dataset was used to fine-tune a newly initialised PICO entity recogniser. Then, we repeat the prediction, pseudo data generation, data selection and supervised fine-tuning procedures, until no more unlabelled records with average prediction probabilities larger than the threshold are identified. Note in every data enlarging step, newly included silver records are split into a training set (80%) and a validation set (20%), then combined with existing gold training records (initially 320 records) and gold validation records (initially 40 records), respectively. This guarantees that the initial gold validation set is only ever used for validation. The original gold test set is used for the final evaluation. All experiments were conducted using an Ubuntu machine with a 16-core CPU.

## Results

The results of the PICO sentence classification models on the test set (425 sentences) are shown in Table 2 (see validation performance in Table 5 in Appendix). All BERT models achieve an F1 score greater than 80% regardless of the pre-training corpus used, and PubMedBERT trained on PubMed abstracts achieves the highest F1 score of 85.4%. Biomedical-domain BERT improves F1 score by 4% compared with general-domain BERT, and BERT with pure biomedical-domain pre-training (two PuBMedBERT) can identify more PICO sentences than BERT with general pre-training (BERT-base) or mixed-domain pre-training (BioBERT), as recall increased by 7%. Therefore, we selected BERT trained on PubMed abstracts as the best PICO sentence classifier for self-training experiments and prediction.

**Table 2:** Performance of PICO sentence classification by BERT with different pre-trained weights on the test set

	F1	Recall	Precision
BERT-base	80.6	81.4	82.1
BioBERT	84.3	81.0	90.0
PubMedBERT-abs	85.4	88.4	85.0
PubMedBERT-full	84.2	87.1	83.8

For PICO entity recognition, for each model, we used settings which achieved the best performance on the validation set and then evaluated these on the test set (40 truncated abstracts). As Table 3 shows, the BERT models (BERT, BERT-CRF, BERT-BiLSTM-CRF) outperformed the LSTM models (BiLSTM, BiLSTM-CRF), with F1 scores improved by between 3 and 27%. The use of a CRF layer improves the F1 score in BiLSTM by 14% but does not enhance performance in BERT models. Compared with the benefit of the large-scale pre-trained domain knowledge, the advantage of the CRF layer might therefore be trivial. Within BERT models, biomedical BERT models improve F1 by at least 4% compared to the general BERT, and the difference between the three biomedical pre-trained weights is not obvious. We selected PuBMedBERT pre-trained on PubMed abstracts and full texts as the best PICO entity recogniser based on the validation results (see Table 6 in Appendix), and the test performance by each PICO entity is reported in Table 4 (‘original scores’). The F1 score for identifying Species is 98%. This entity has a limited number of potential responses, so their identification is not complicated. For Intervention and Outcome, the performance is satisfactory, with F1 around 70%. F1 scores of Strain and Induction are 63% and 49%, respectively, so there remains room for improvement. The F1 score for identifying the Comparator is only 16%, which may be due to the relative lack of Comparator instances in the training corpus and unclear boundaries in the definition of comparator and interventions in some complicated manuscripts. For instance, a manuscript may describe two experiments, and what is an intervention in the first may become a comparator in the second.

**Table 3:** Overall performance of the PICO entity recognition models on the test set

Model	Weight	F1	Recall	Precision
BiLSTM	–	43.5	38.1	50.6
BiLSTM-CRF	–	57.9	54.7	61.6
BERT	Base	61.3	66.3	57.1
	BioBERT	65.4	69.8	61.5
	PubMed-abs	70.1	73.2	67.3
	PubMed-full	69.9	73.4	66.7
BERT-CRF	Base	62.1	67.2	57.8
	BioBERT	66.5	70.1	63.3
	PubMed-abs	68.0	71.5	64.9
	PubMed-full	67.5	70.9	64.5
BERT - BiLSTM - CRF	Base	64.6	69.5	60.3
	BioBERT	68.3	71.2	65.6
	PubMed-abs	67.2	70.8	64.0
	PubMed-full	68.5	72.6	64.8

**Table 4:** Entity-level performance of PubMedBERT on the gold test set. Original scores refer to the performance of the model before self-training; self-training scores refer to the performance of the model at the best iteration (6th iteration) of self-training. ‘R’ and ‘P’ refer to recall and precision, respectively

	Original scores			Self-training scores		
	F1	R	P	F1	R	P
**Comparator**	16.0	10.0	40.0	48.5	40.0	61.5
**Induction**	49.1	50.6	47.7	48.0	49.4	46.6
**Intervention**	70.2	76.1	65.2	69.8	74.6	65.6
**Outcome**	65.4	70.6	60.9	66.9	70.6	63.6
**Species**	98.1	100.0	96.4	98.1	100.0	96.4
**Strain**	63.4	72.2	56.5	70.0	77.8	63.6
**Overall**	69.9	73.4	66.7	71.0	74.0	68.2

**Fig. 4:**
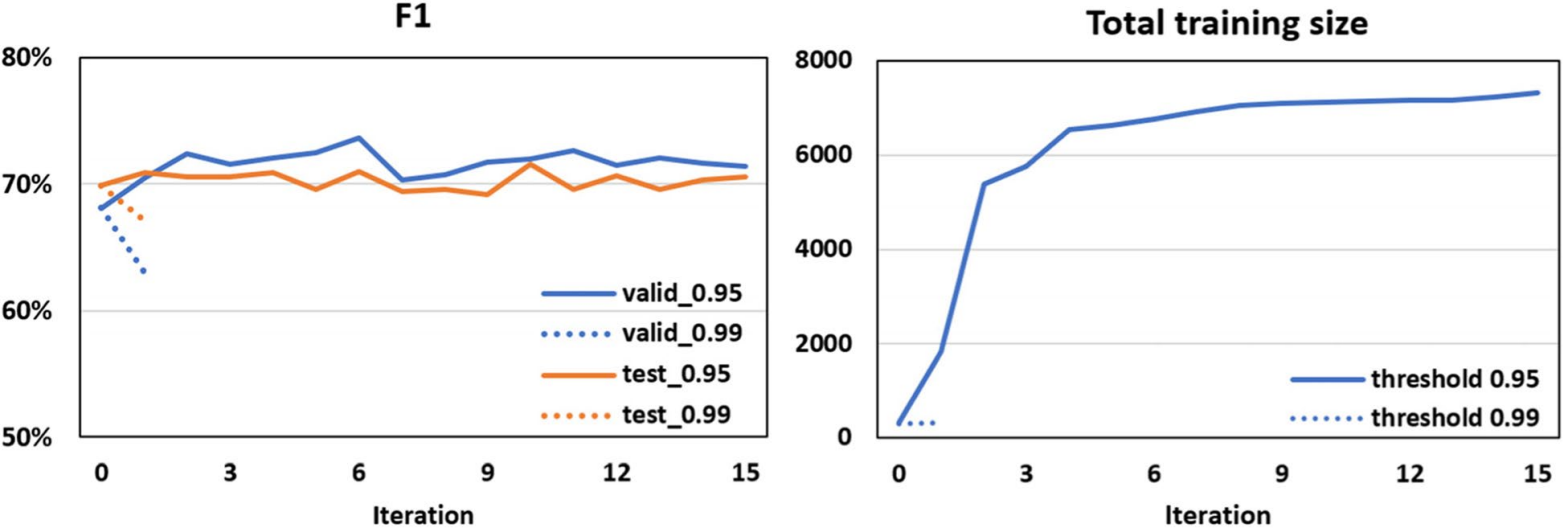
Performance of PubMedBERT for PICO entity recognition using self-training

In self-training experiments, we used the best PICO sentence classifier (BERT pre-trained on PubMed abstracts) to remove non-PICO sentences for unlabelled data and the best PICO entity recogniser (BERT pre-trained on PubMed abstracts and full texts) to identify PICO phrases and calculate prediction scores across all tokens in each individual text. We explore two thresholds (0.95, 0.99) for record selection, and the results are reported in Fig. 4. When the threshold is 0.99, no more silver records are included in the training set beyond the first iteration, and self-training did not improve performance. When the threshold is 0.95, the performance fluctuates and the best F1 score is improved by 5% and 1% on the gold validation set and test set, respectively, achieved at the sixth iteration step. We terminated the training programme after 15 iterations because the training size tends to saturate and the improvement of performance is very limited. For specific PICO entities, the main improvement using self-training was for F1 scores for Comparator and Strain, which increased by 32% and 7%, respectively (‘self-training scores’ in Table 4).

**Fig. 5:**
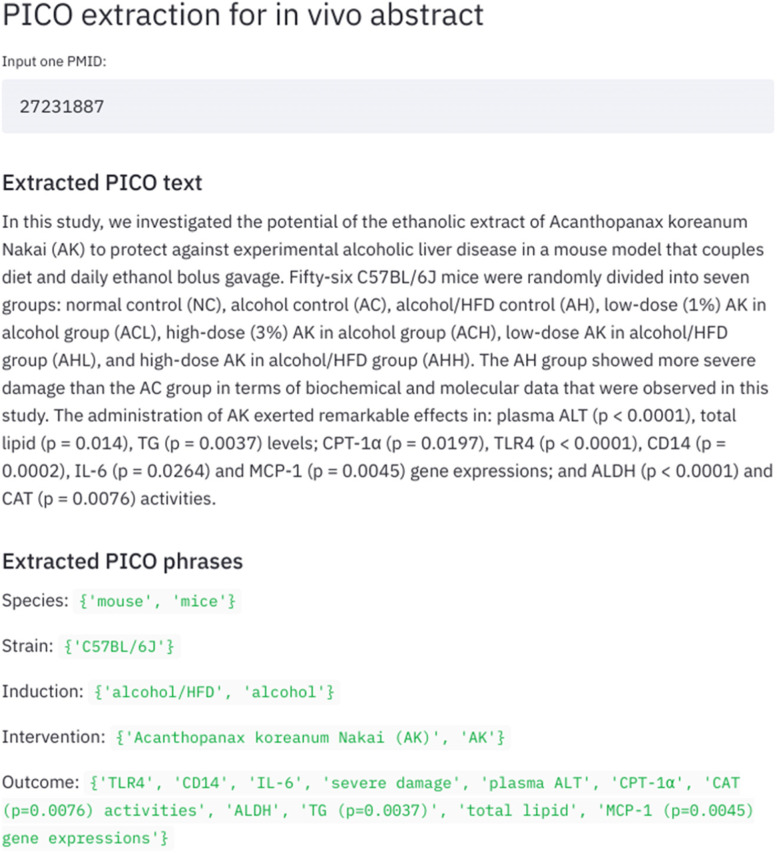
The visualisation of the Streamlit app

We have developed an interactive application via Streamlit^3^ for potential use (see Fig. 5). When the user inputs the PMID from the PubMed Open Access Subset, the app will call the PubMed Parser [33] to return its title and abstract. The background sentence model classifies and removes non-PICO sentences, and then the entity recogniser identifies the PICO phrases from those PICO sentences. This can give a quick overview of the PICO elements of an experimental study.

## Discussion

In this work, we show the possibilities of automated PICO sentence classification and PICO entity recognition in abstracts describing preclinical animal studies. For sentence classification, BERT models with different pre-trained weights have generally good performance (F1 over 80%), and biomedical BERT (BioBERT or PubMedBERT) have slightly better performance than general BERT. For PICO entity recognition, all BERT models outperform BiLSTM with or without a CRF layer, with the improvement of F1 ranging from 3 to 27%. It is unnecessary to use a more complicated structure based on BERT, as the results of BERT, BERT-BiLSTM and BERT-BiLSTM-CRF do not differ greatly, but the latter two bring a cost in longer training time and resources. Within LSTM-based models, adding a CRF layer is beneficial, where recall is increased by 16% and precision is increased by 9%. The training time of LSTM-based models is much shorter than fine-tuning BERT, and this could be a quick alternative solution when computing resources are limited, at the cost of reduction of performance by 3% and 12% compared to the general BERT and PubMedBERT, respectively. The self-training approach helps to identify more comparators and strains but does not help much with the overall performance. By entity levels, F1 scores are generally good for identifying Species (over 80%); satisfactory for Intervention, Outcome and Strain (around or over 70%); and acceptable for Induction and Comparator (around 50%).

We randomly selected 10 abstracts from the test set to investigate the modules of PICO sentence classification and PICO entity recognition. The PICO sentence classifier works well in most cases as the performance demonstrates. The main error comes from the judgements of the definition of PICO sentences in the annotation process. In some cases, the first introduction sentence explains a PICO phrase and its abbreviation, and the following texts mention only the abbreviation word. We annotated that sentence as a PICO sentence because our original purpose is to enable the model to extract the full name which indicates the meaning of the abbreviation word. However, the model did not recognise it as a PICO sentence because most general introduction sentences in an abstract do not describe the actual experimental procedures. In other cases, the model extracts some sentences describing the purpose of the study, explaining the research findings or discussing the background mechanism as PICO sentences. Those sentences are often placed before the method sentences or after the result sentences, and some of them mention PICO phrases but do not explicitly describe the experimental procedures or the specific outcomes and interventions. Considering the functionality and relative position of those sentences in the entire abstract, we did not annotate them as PICO sentences, but it is ambiguous in the model training. The ambiguity of PICO sentence definition in the annotation process and the accuracy of the PICO sentence classifier may further affect the performance of the PICO entity recogniser.

In the error analysis for PICO entity recognition, one issue is the boundary of PICO phrases. For example, an outcome phrase is ‘level of plasma corticosterone’, but our model extracts ‘plasma corticosterone’. In another example, the outcome annotations are ‘VEGF mRNA’ and ‘VEGF protein’, but our model combines two text spans into one phrase ‘VEGF mRNA and VEGF protein’, which reduces the scores calculated in the validation process but does not affect users to obtain information from the output. The second issue is we did not annotate summarised or indirect phrases but our model extracts those types of outcomes. For example, in the sentence ‘Met-knockdown reduced tumour burden correlating with decreased cell survival and tumour angiogenesis, with minimal effect on cell growth’, our annotation of Outcome includes ‘cell survival’, ‘tumour angiogenesis’ and ‘cell growth’ but excludes ‘tumour burden’ which is extracted by the model.

One limitation of our work is that the training corpus is at the level of the abstract, but some PICO elements in preclinical animal studies are often not described in the abstract. This limits the usefulness of our applications, and we cannot transfer it to full-text identification without further evaluation. Of note, this same limitation applies to manual approaches to identifying PICO elements based on the abstract alone. In a related literature, we have shown, for instance, that manual screening for inclusion based on TiAb has substantially lower sensitivity than the manual screening of full texts (https://osf.io/nhjeg). Another limitation is that the amount of training, validation and test data is not adequate. Although our best models do not show very inconsistent results between validation and test set (except for ‘Comparator’), the conclusions may still be biased using a small dataset. Previous studies show that self-training can propagate both knowledge and error from high confidence predictions on unlabelled samples [34] and that training from larger annotated corpora may reduce the error propagation and boost performance. Large datasets also provide possibilities for exploring more complicated models which are proved effective in other tasks.

In future work, we will evaluate our PICO sentence classification and entity recognition models in some full-text publications, to observe any heuristic implications. We will also evaluate the existing clinical PICO extraction tools on preclinical text to identify interventions and outcomes because these two categories may be more similar in preclinical and clinical studies than other PICO elements. Some automation tools developed for clinical PICO extraction could be evaluated in preclinical publications. For example, Trialstreamer [35] could be used to identify interventions and outcomes in preclinical experiments. As the training corpora for clinical PICO are relatively larger and in more standard forms, we think that training using a combined preclinical/clinical corpus may yield better performance.

## Conclusions

We demonstrate a workflow for PICO extraction in preclinical animal text using LSTM- and BERT-based models. Without feature engineering, BERT pre-trained on PubMed abstracts is optimal for both PICO sentence classification, and BERT pre-trained on PubMed abstracts and full texts is optimal for PICO entity recognition tasks in preclinical abstracts. PICO entities including Intervention, Outcome, Species and Strain have acceptable precision and recall (around or over 70%), while Comparator and Induction have less satisfactory scores (around 50%). We encourage the collection of a more standard PICO annotation corpus and the use of natural language processing models for PICO extraction in preclinical animal studies, which may achieve better results for publications retrieval, reduce the workflow of preclinical systematic reviews and narrow the gap between preclinical and clinical research. The datasets, code and the optimal trained models supporting the current study are publicly available in the Preclinical PICO extraction repository, https://osf.io/2dqcg.

## Data Availability

The datasets supporting the current study are available in the Preclinical PICO extraction repository, https://osf.io/2dqcg.

## Appendix

Tables 5, 6 and 7

**Table 5:** Performance of PICO sentence classification by BERT with different pre-trained weights on the validation set

	F1	Recall	Precision
BERT-base	86.6	87.7	87.2
BioBERT	87.7	89.6	88.1
PubMedBERT-abs	89.3	91.3	89.1
PubMedBERT-full	85.8	89.3	84.6

**Table 6:** Overall performance of PICO entity recognition models on the validation set

Model	Weight	F1	Recall	Precision
BiLSTM	–	41.7	44.2	39.5
BiLSTM-CRF	–	58.8	56.9	61.0
BERT	Base	56.0	62.7	50.6
	BioBERT	64.2	69.8	59.4
	PubMed-abs	65.0	70.5	60.2
	PubMed-full	68.1	73.0	63.8
BERT-CRF	Base	57.7	62.9	53.3
	BioBERT	65.1	70.0	60.9
	PubMed-abs	65.5	70.9	60.9
	PubMed-full	68.0	72.8	63.7
BERT - BiLSTM - CRF	Base	60.8	66.4	56.1
	BioBERT	66.0	70.0	62.5
	PubMed-abs	68.1	73.3	63.5
	PubMed-full	68.0	72.8	63.8

**Table 7:** Entity-level performance of PubMedBERT on the gold validation set. Original scores refer to the performance of the model before self-training; self-training scores refer to the performance of the model at the best iteration (6th iteration) of self-training. ‘R’ and ‘P’ refer to recall and precision, respectively

	Original scores			Self-training scores		
	F1	R	P	F1	R	P
**Comparator**	33.3	66.7	22.2	80.0	66.7	100.0
**Induction**	46.2	50.9	42.3	45.7	40.7	52.2
**Intervention**	67.3	69.6	65.2	69.6	75.0	64.9
**Outcome**	61.5	68.0	56.2	69.9	73.5	66.7
**Species**	96.4	99.1	93.9	96.4	99.1	93.9
**Strain**	80.0	80.0	80.0	90.9	100.0	83.3
**Overall**	68.1	73.0	63.8	73.6	76.4	71.0

## Abbreviations

BERT: Bidirectional encoder representations from transformers
BiLSTM: Bidirectional long-short term memory
BIO: Beginning-Inside-Outside tagging format
CRF: Conditional random field
NER: Named entity recognition
PICO: Population, Intervention, Comparator and Outcome
tsv: Tab-separated values

## Footnotes

1
https://www.ncbi.nlm.nih.gov/pmc/tools/openftlist

2
https://www.tagtog.net

3
https://streamlit.io/
